# Comparative effects of soy protein concentrate, enzyme-treated soybean meal, and fermented soybean meal replacing animal protein supplements in feeds on growth performance and intestinal health of nursery pigs

**DOI:** 10.1186/s40104-023-00888-3

**Published:** 2023-07-02

**Authors:** Zixiao Deng, Marcos Elias Duarte, So Young Kim, Yunil Hwang, Sung Woo Kim

**Affiliations:** 1 Department of Animal Science, North Carolina State University, 116 Polk Hall, Campus Box 7621, Raleigh, NC 27695 USA; 2 CJ Cheiljedang, Seoul, 04560 Korea

**Keywords:** Enzyme-treated soybean meal, Fermented soybean meal, Intestinal health, Nursery pigs, Soy protein concentrate

## Abstract

**Background:**

Soy protein supplements, with high crude protein and less antinutritional factors, are produced from soybean meal by different processes. This study evaluated the comparative effects of various soy protein supplements replacing animal protein supplements in feeds on the intestinal immune status, intestinal oxidative stress, mucosa-associated microbiota, and growth performance of nursery pigs.

**Methods:**

Sixty nursery pigs (6.6 ± 0.5 kg BW) were allotted to five treatments in a randomized complete block design with initial BW and sex as blocks. Pigs were fed for 39 d in 3 phases (P1, P2, and P3). Treatments were: Control (CON), basal diet with fish meal 4%, 2%, and 1%, poultry meal 10%, 8%, and 4%, and blood plasma 4%, 2%, and 1% for P1, P2, and P3, respectively; basal diet with soy protein concentrate (SPC), enzyme-treated soybean meal (ESB), fermented soybean meal with *Lactobacillus* (FSBL), and fermented soybean meal with *Bacillus* (FSBB), replacing 1/3, 2/3, and 3/3 of animal protein supplements for P1, P2, and P3, respectively. Data were analyzed using the MIXED procedure in SAS 9.4.

**Results:**

The SPC did not affect the BW, ADG, and G:F, whereas it tended to reduce (*P* = 0.094) the ADFI and tended to increase (*P* = 0.091) crypt cell proliferation. The ESM did not affect BW, ADG, ADFI, and G:F, whereas tended to decrease (*P* = 0.098) protein carbonyl in jejunal mucosa. The FSBL decreased (*P* < 0.05) BW and ADG, increased (*P* < 0.05) TNF-α, and *Klebsiella* and tended to increase MDA (*P* = 0.065) and IgG (*P* = 0.089) in jejunal mucosa. The FSBB tended to increase (*P* = 0.073) TNF-α, increased (*P* < 0.05) *Clostridium* and decreased (*P* < 0.05) *Achromobacter* and alpha diversity of microbiota in jejunal mucosa.

**Conclusions:**

Soy protein concentrate, enzyme-treated soybean meal, and fermented soybean meal with *Bacillus* could reduce the use of animal protein supplements up to 33% until 7 kg body weight, up to 67% from 7 to 11 kg body weight, and entirely from 11 kg body weight without affecting the intestinal health and the growth performance of nursery pigs. Fermented soybean meal with *Lactobacillus*, however, increased the immune reaction and oxidative stress in the intestine consequently reducing the growth performance.

## Background

At weaning, the change from highly digestible sow milk to plant-based solid diets, living environment, and social hierarchy leads to the most stressful period for pigs [1, 2]. The stress commonly causes intestinal and immunological dysfunction leading to impaired health and growth of nursery pigs [3]. Animal proteins are usually added to the diet for nursery pigs to alleviate the weaning stress by stimulating feed intake and improving intestinal health due to the highly digestible protein and functional compounds [4, 5]. However, there are some concerns about using animal protein supplements in nursery diets, including the cost, availability, and safety issues [6]. Compared with animal proteins, soybean meal is a more affordable and steady protein source that is most widely used in the swine diet [7]. However, the use of soybean meal is limited especially for feeding nursery pigs due to its antinutritional factors including soy allergens, lectins, trypsin inhibitors, and flatulence-producing oligosaccharides [8]. These can impair the growth and intestinal health of pigs [9, 10]. Therefore, several methods have been attempted to eliminate the antinutritional factors in soybean meal.

In order to overcome the limitations, such as antinutritional factors, soybean meal can be further processed. Soy protein concentrate is produced by ethanol extractions of soybean meal to remove soluble carbohydrates, reduce soy allergens, and keep a relatively high crude protein content than soybean meal [11, 12]. Enzyme treatment involves treating soybean meal with a proprietary mixture of enzymes to hydrolyze soluble carbohydrates and antinutritional factors [13]. The fermentation process involves treating soybean meal with different microorganisms to reduce antinutritional factors because of the enzymes secreted by microorganisms [8, 14]. Besides the reduction of antinutritional factors in soybean meal, the process of enzymatic hydrolysis and microbial fermentation also can increase the proportions of small peptides, which can improve the utilization of dietary proteins by nursery pigs when their endogenous protease secretion is limited [15–17]. Several studies have indicated that processed soybean products can improve the growth performance of nursery pigs compared to soybean meal [18–20]. In addition, studies have suggested that these processed soy products have the potential to partly replace the use of animal protein in the diets of nursery pigs [12, 21, 22].

Based on previous findings, it was hypothesized that soy protein concentrate has equal quality compared with enzyme-treated soy protein and fermented soybean meal to replace animal protein supplements in the diets of nursery pigs. To test the hypothesis, the objective of this study was to evaluate the comparative effects of soy protein concentrate, enzyme-treated soybean meal, and fermented soybean meal replacing animal protein supplements in feeds on intestinal immune status, intestinal oxidative stress, mucosa-associated microbiota, and growth performance of nursery pigs.

## Materials and methods

The Institutional Animal Care and Use Committee of North Carolina State University (Raleigh, NC, USA) revised and approved the protocol used for this study. The animal experiment was conducted at the North Carolina State University Metabolism Educational Unit (Raleigh, NC, USA).

### Allergenic proteins in soy products

The β-conglycinin ELISA Kit (BA-UBT001, Unibiotest, Wuhan, China) and Glycinin ELISA Kit (BA-UBT002, Unibiotest) were used to measure the content of β-conglycinin and glycinin in the soy protein supplements as previously described by Deng et al. [12]. The soy protein supplements included soybean meal (North Carolina State University Feed Mill Education Unit, Raleigh, NC, USA), soy protein concentrate (CJ Selecta, Araguari, MG, Brazil), enzyme-treated soybean meal (Hamlet Protein Inc., Findlay, OH, USA), fermented soybean meal with *Lactobacillus* (Purina Animal Nutrition, Shoreview, MN, USA), and fermented soybean meal with *Bacillus* (CJ Bio, Seoul, Korea). The soy protein samples were ground and weighed (0.3 g) to mix with 30 mL sample extractant. The mixed samples were vigorously shaken for 16 h at 25 °C and then centrifuged at 4,000 × *g* for 5 min to get supernatant. Extracted samples and standards (50 µL) were added in the plate and antibody solution (50 µL) were added. The plate was gently shaken to mix these two reagents and incubated at 37 °C for 30 min. Next, the plate was washed by wash solution 4 times and 100 µL horseradish peroxidase (HRP) conjugate enzyme was added in each well to incubate at 37 °C for 30 min. The plate was washed then the mixture of chromogen solution A and B (100 µL) were added in each well to incubate at 37 °C for 15 min. At the end, 50 µL stop solution was added in each well and the plate was read within 15 min. The absorbance was measured by the spectrophotometer (Synergy HT, BioTek Instruments, Winooski, VT, USA) at 450 and 630 nm wavelengths. The concentrations of β-conglycinin and glycinin were expressed as mg/g (Table 1).

**Table 1:** Concentration of soy antigens in soy products

Item	SBM^a^	SPC^a^	ESB^a^	FSBL^a^	FSBB^a^
Glycinin, mg/g	112.6 ± 24.3	< 0.1 ± 0.0	0.2 ± 0.0	164.5 ±12.0	66.9 ± 13.6
β-conglycinin, mg/g	125.0 ± 10.2	0.1 ± 0.0	0.3 ± 0.0	137.9 ± 10.6	21.0 ± 0.1

^a^SBM: soybean meal, North Carolina State University Feed Mill Education Unit, Raleigh, NC, USA; SPC: X-Soy 200, CJ Selecta, Araguari, MG, Brazil; ESB: HP 300, Hamlet Protein Inc., Findlay, OH, USA; FSBL: FerMex 200, Purina Animal Nutrition, Shoreview, SD, USA; FSBB: Soytide, CJ Bio, Jung-gu, Seoul, Korea

### Animal, design, and diets

Sixty newly-weaned pigs (30 barrows and 30 gilts) weaned at 21 days of age with 6.6 ± 0.5 kg of initial body weight (BW) were allotted to four dietary treatments in a randomized complete block design. Sex and initial BW were considered as blocking criteria. Pigs were purchased from Kilpatrick Hog Farm (Magnolia, NC, USA) and individually housed in pens with a waterer and feeder. All pens (1.50 m × 0.74 m) were located in the same room. Pigs were fed for 39 d in 3 phases (P1 for 7 d [from wean to 7 kg BW]; P2 for 13 d [from 7 to 11 kg BW]; P3 for 19 d [from 11 to 25 kg BW]). Dietary treatments were control (CON): basal diet with fish meal (at 4%, 2%, and 1% for P1, P2, and P3 respectively), poultry meal (at 10%, 8%, and 4% for P1, P2, and P3 respectively), and blood plasma (at 4%, 2%, and 1% for P1, P2, and P3 respectively); SPC: basal diet with soy protein concentrate replacing animal protein supplements (at 1/3, 2/3, and 3/3 for P1, P2, and P3, respectively), ESB: basal diet with enzyme-treated soybean meal replacing animal protein supplements (at 1/3, 2/3, and 3/3 for P1, P2, and P3, respectively), FSBL: basal diet with fermented soybean meal with *Lactobacillus* replacing animal protein supplements (at 1/3, 2/3, and 3/3 for P1, P2, and P3, respectively); and FSBB: basal diet with fermented soybean meal with *Bacillus* replacing animal protein supplements (1/3, 2/3, and 3/3 for P1, P2, and P3, respectively). The replacement of protein supplements was gradually increased (1/3 to 3/3) with the progress of phase feeding (P1 to P3) based on the outcomes of our previous study [12].

All nutrients in the experimental diets met or were slightly higher than the requirement suggested by NRC [23]. An indigestible external marker (0.4% titanium dioxide) was mixed with all experimental diets and fed to pigs from d 32 to 39 of the experiment. The composition of the experimental diets are shown in Table 2. The Feed Mill Educational Unit of North Carolina State University (Raleigh, NC, USA) produced all experimental diets. Samples of treatment diets were ground and sent to the North Carolina Department of Agriculture and Consumer Services to analyze the nutritional compositions (Raleigh, NC, USA).

**Table 2:** Composition of experimental diets for experiment

Item	Phase 1					Phase 2					Phase 3				
	CON	SPC^a^	ESB^a^	FSBL^a^	FSBB^a^	CON	SPC	ESB	FSBL	FSBB	CON	SPC	ESB	FSBL	FSBB
Ingredient^b^, %															
Corn, yellow	28.79	27.78	27.96	27.60	28.13	40.39	39.48	39.65	39.24	39.82	62.58	61.85	61.92	61.62	62.02
Whey permeate	24.00	24.00	24.00	24.00	24.00	15.00	15.00	15.00	15.00	15.00	5.00	5.00	5.00	5.00	5.00
Cookie meal	10.00	10.00	10.00	10.00	10.00	10.00	10.00	10.00	10.00	10.00	0.00	0.00	0.00	0.00	0.00
Soybean meal (48% CP)	16.00	16.00	16.00	16.00	16.00	19.00	19.00	19.00	19.00	19.00	23.00	23.00	23.00	23.00	23.00
Poultry meal	10.00	6.67	6.67	6.67	6.67	8.00	2.67	2.67	2.67	2.67	4.00	0.00	0.00	0.00	0.00
Fish meal (63% CP)	4.00	2.67	2.67	2.67	2.67	2.00	0.67	0.67	0.67	0.67	1.00	0.00	0.00	0.00	0.00
Blood plasma	4.00	2.67	2.67	2.67	2.67	2.00	0.67	0.67	0.67	0.67	1.00	0.00	0.00	0.00	0.00
X-Soy200	0.00	6.50	0.00	0.00	0.00	0.00	7.80	0.00	0.00	0.00	0.00	6.00	0.00	0.00	0.00
HP300	0.00	0.00	6.00	0.00	0.00	0.00	0.00	7.20	0.00	0.00	0.00	0.00	5.54	0.00	0.00
Fermex200	0.00	0.00	0.00	7.09	0.00	0.00	0.00	0.00	8.51	0.00	0.00	0.00	0.00	6.54	0.00
Soytide	0.00	0.00	0.00	0.00	6.62	0.00	0.00	0.00	0.00	7.95	0.00	0.00	0.00	0.00	6.11
Poultry fat	1.30	1.50	1.70	1.00	1.00	1.30	1.80	2.10	1.20	1.30	1.00	1.30	1.60	0.90	1.00
*L*-Lys HCl	0.52	0.53	0.54	0.54	0.54	0.51	0.52	0.53	0.53	0.53	0.42	0.43	0.43	0.43	0.43
*L*-Met	0.25	0.24	0.25	0.25	0.23	0.21	0.20	0.21	0.21	0.18	0.14	0.13	0.14	0.14	0.12
*L*-Thr	0.17	0.11	0.17	0.17	0.15	0.15	0.09	0.16	0.16	0.13	0.12	0.07	0.12	0.12	0.10
*L*-Trp	0.02	0.01	0.01	0.02	0.01	0.01	0.00	0.00	0.02	0.00	0.00	0.00	0.00	0.00	0.00
*L*-Val	0.00	0.00	0.00	0.00	0.00	0.00	0.00	0.01	0.02	0.01	0.00	0.00	0.00	0.00	0.00
Dicalcium phosphate	0.00	0.05	0.05	0.00	0.00	0.15	0.65	0.65	0.60	0.60	0.54	0.90	0.90	0.90	0.90
Limestone, ground	0.30	0.62	0.66	0.67	0.66	0.63	0.80	0.83	0.85	0.82	0.80	0.92	0.95	0.95	0.92
Zinc oxide	0.25	0.25	0.25	0.25	0.25	0.25	0.25	0.25	0.25	0.25	0.00	0.00	0.00	0.00	0.00
Salt	0.22	0.22	0.22	0.22	0.22	0.22	0.22	0.22	0.22	0.22	0.22	0.22	0.22	0.22	0.22
Mineral premix^c^	0.15	0.15	0.15	0.15	0.15	0.15	0.15	0.15	0.15	0.15	0.15	0.15	0.15	0.15	0.15
Vitamin premix^d^	0.03	0.03	0.03	0.03	0.03	0.03	0.03	0.03	0.03	0.03	0.03	0.03	0.03	0.03	0.03
Calculated composition (as-is)															
Dry matter, %	91.3	91.4	91.2	91.0	91.0	90.7	90.8	90.6	90.4	90.4	89.7	89.8	89.6	89.4	89.4
ME, kcal/kg	3,454	3,454	3,449	3,452	3,449	3,435	3,437	3,435	3,436	3,437	3,373	3,373	3,374	3,373	3,376
Crude protein, %	24.6	24.4	23.8	23.8	24.1	22.5	21.9	21.2	21.2	21.5	20.9	20.5	19.9	19.9	20.1
SID^e^ Lys, %	1.50	1.50	1.50	1.50	1.50	1.35	1.35	1.35	1.35	1.35	1.23	1.23	1.23	1.23	1.23
SID Met+Cys, %	0.82	0.82	0.82	0.82	0.82	0.74	0.74	0.74	0.74	0.74	0.68	0.68	0.68	0.68	0.68
SID Trp, %	0.25	0.25	0.25	0.25	0.25	0.22	0.22	0.22	0.22	0.22	0.20	0.21	0.21	0.20	0.21
SID Thr, %	0.88	0.88	0.88	0.88	0.88	0.79	0.79	0.79	0.79	0.79	0.73	0.73	0.73	0.73	0.73
Ca, %	0.85	0.85	0.85	0.85	0.85	0.80	0.80	0.80	0.80	0.80	0.70	0.70	0.70	0.70	0.70
STTD P^f^, %	0.50	0.45	0.45	0.45	0.45	0.40	0.40	0.40	0.40	0.40	0.33	0.33	0.33	0.33	0.33
Total P, %	0.73	0.67	0.66	0.65	0.65	0.63	0.62	0.62	0.60	0.61	0.58	0.57	0.57	0.56	0.56
Glycinin^g^, mg/g	18.0	18.0	18.0	29.7	22.4	21.4	21.4	21.4	35.4	26.7	25.9	25.9	25.9	36.7	30.0
β-Conglycinin^h^, mg/g	20.0	20.0	20.0	29.8	21.4	23.8	23.8	23.8	35.5	25.4	28.8	28.8	28.8	37.8	30.0
Analyzed composition (as-is)															
Dry matter, %	91.7	91.6	91.8	91.3	91.5	90.4	90.3	90.8	90.4	90.9	88.4	88.4	88.5	88.3	88.3
Crude protein, %	24.6	24.0	23.3	23.1	23.8	21.3	21.2	20.8	19.7	21.1	20.5	19.9	19.6	19.0	18.8
Neutral detergent fiber, %	8.1	7.9	7.8	7.6	7.6	9.1	8.6	8.6	8.3	8.2	9.8	9.6	9.0	8.8	9.4
Acid detergent fiber, %	3.0	3.2	3.3	3.0	3.5	3.5	3.9	3.7	3.7	3.7	4.1	3.9	4.0	4.0	4.1
Ca, %	0.77	0.78	0.86	0.80	0.81	0.76	0.74	0.79	0.77	0.79	0.68	0.67	0.73	0.74	0.62
Total P, %	0.71	0.66	0.66	0.65	0.66	0.62	0.62	0.62	0.58	0.60	0.57	0.58	0.55	0.53	0.54

^a^SPC, ESB, FSBL, and FSBB: basal diet with soy protein concentrate (X-Soy 200, CJ Selecta, Araguari, MG, Brazil), enzyme-treated soybean meal (HP 300, Hamlet Protein Inc., Findlay, OH, USA), fermented soybean meal with *Lactobacillus* (FerMex 200, Purina Animal Nutrition, Shoreview, SD, USA), and fermented soybean meal with *Bacillus* (Soytide, CJ Bio, Seoul, Korea), replacing 1/3, 2/3, 3/3 of animal protein supplements in P1, P2, P3, respectively

^b^The nutritional composition of corn, soybean meal, fish meal, blood plasma, and poultry fat were from NRC [23]; the nutritional composition of poultry meal were from Rojas and Stein [24]; the nutritional composition of whey permeate, cookie meal, X-Soy200, HP300, Fermex200, and Soytide were from analyzed values (International Ingredient Corporation, Fenton, MO, USA; Darling Ingredients, Irving, TX, USA; CJ Selecta, Araguari, MG, Brazil; Hamlet Protein Inc., Findlay, OH, USA; Purina Animal Nutrition, Shoreview, SD, USA; CJ Bio, Seoul, Korea); the nutritional composition of all amino acids were from analyzed values (CJ Bio, Seoul, Korea); the nutritional composition of dicalcium phosphate, limestone, salt, mineral premix, and vitamin premix were from analyzed values (North Carolina State University Feed Mill Education Unit, Raleigh, NC, USA); the nutritional composition of zinc oxide was from analyzed values (Zinc Nacional, Monterrey, N.L., Mexico)

^c^The trace mineral premix provided per kilogram of complete diet: 33 mg of Mn as manganous oxide, 110 mg of Fe as ferrous sulfate, 110 mg of Zn as zinc sulfate, 16.5 mg of Cu as copper sulfate, 0.30 mg of I as ethylenediamine dihydroiodide, and 0.30 mg of Se as sodium selenite

^d^The vitamin premix provided per kilogram of complete diet: 6,614 IU of vitamin A as vitamin A acetate, 992 IU of vitamin D_3_, 19.8 IU of vitamin E, 2.64 mg of vitamin K as menadione sodium bisulfate, 0.03 mg of vitamin B_12_, 4.63 mg of riboflavin, 18.52 mg of *D*-pantothenic acid as calcium pantothenate, 24.96 mg of niacin, and 0.07 mg of biotin

^e^*SID* Standardized ileal digestibility

^f^*STTD P* Standardized total tract digestible phosphorus

^g^The concentration of glycinin in the diets was calculated based on the analyzed glycinin of soy protein supplements

^h^The concentration of β-conglycinin in the diets was calculated based on the analyzed β-conglycinin of soy protein supplements

### Growth performance and fecal score

The growth performance including average BW, ADG, ADFI, and G:F were calculated based on the BW and feed intake obtained on d 0, 7, 20, and 39. Fecal scores were evaluated daily by the same person considering the following scale: very hard and dry stool (score 1), firm stool (score 2), normal stool (score 3), loose stool (score 4), and watery stool with no shape (score 5) as described by Cheng et al. [25].

### Sample collection

At the end of the study, euthanasia of pigs was conducted by the penetration of a captive bolt to the head followed by exsanguination [26]. The entire gastrointestinal tract was removed to collect samples. Ileal digesta (50 cm anterior to the ileocecal junction to the end of the ileum) was collected and put on the ice, then stored at −20 °C for further analysis. To evaluate morphology, mid-jejunum segments (5 cm) were washed with 0.9% saline solution and then placed in a 50-mL falcon tube containing 10% buffered formaldehyde. Mid-jejunal mucosal samples were scraped off with a glass slide and collected in 2-mL tubes, then promptly frozen in liquid nitrogen. The mucosal samples were transferred to the freezer at −80 °C for further DNA extraction and intestinal oxidative stress and immune status analysis.

### Oxidative stress and immune status

The mucosal samples were processed following Holanda and Kim [27]. One gram of jejunal mucosa was weighed and then suspended in 1 mL of phosphate-buffered saline (PBS) in a 5-mL tube. A tissue homogenizer (Tissuemiser; Thermo Fisher Scientific Inc, Waltham, MA, USA) was used to homogenize the suspended mucosal samples. The homogenized samples were transferred into a new tube and centrifuged (13,000 × *g* for 10 min), then six aliquots of the supernatant were pipetted off and kept at −80 °C.

Colorimetric assays kits, commercially available, were used to measure the total protein, tumor necrosis factor-alpha (TNF-α), interleukin 8 (IL-8), immunoglobulin A (IgA), immunoglobulin G (IgG), malondialdehyde (MDA), and protein carbonyl following the instructions of the manufacturers. The OD value was measured using the spectrophotometer (Synergy HT, BioTek Instruments) and software (Gen5 Data Analysis Software, BioTek Instruments). According to the OD value of the respective standards, the concentration of each parameter was calculated.

The total protein concentration was determined using the Pierce BCA Protein Assay Kit (#23225, Thermo Fisher Scientific) following the procedure described by Holanda et al. [28]. The mucosal supernatant was diluted (1:60) in PBS to provide the protein concentration within the proper range (20–2,000 g/mL). The samples and standards (25 µL) were pipetted in microplate well and then 200 µL working reagent was added to each well. The plate was incubated at 37 °C for 30 min. The absorbance was measured at 562 nm wavelength. The total protein concentration was used to further standardize the concentration of other measures in the mucosa.

The concentration of TNF-α in the jejunal mucosa was determined using Porcine TNF--α DuoSet ELISA Kit (#DY690B, R&D Systems; Minneapolis, MN, USA) following the procedure described by Sun et al. [29]. The samples and standards (100 µL) were added in each well of the plate and then 100 µL detection antibody was added with 2 h incubation at room temperature. The Streptavidin-HRP (100 µL) and substrate solution (100 µL) were consecutively used in the analysis. Stop solution (50 µL) was used to stop the reaction. The absorbance was read at 450 nm wavelength and corrected at 570 nm wavelength. The concentration of TNF-α was reported as pg/mg of protein. The Porcine IL-8/CXCL8 DuoSet ELISA Kit (#DY535, R&D Systems) was used to determine the concentration of IL-8 in jejunal mucosa following the procedure described by Moita et al. [30]. The mucosal supernatant was diluted (1:5) using the reagent diluent provided in the kit to reach the proper working range. The samples and standards (100 µL) were added in each well of the plate and then 100 µL detection antibody was added with 2 h incubation at room temperature. The Streptavidin-HRP (100 µL) and substrate solution (100 µL) were consecutively used in the analysis. Stop solution (50 µL) was used to stop the reaction. The absorbance was read at 450 nm wavelength and corrected at 570 nm wavelength and the concentration of IL-8 was reported as pg/mg of protein.

The Pig IgA ELISA Kit and Pig IgG ELISA Kit (E101-102 and E101-104, Bethyl Laboratories, Inc., Montgomery, TX, USA) were used to determine the concentration of IgA and IgG following the procedure described by Holanda et al. [28]. The PBS was used to dilute the mucosal supernatant (1:1,200 and 1:2,400, for IgA and IgG respectively) to reach the working range of the ELISA kits. The samples and standards were added in each and incubated at room temperature for 1 h. Then 100 µL anti-IgA detection antibody was added and incubated at room temperature for 1 h. The HRP solution (100 µL) and 3,3’,5,5’-tetramethylbenzidine (TMB) substrate solution (100 µL) were consecutively used in the analysis. Stop solution (100 µL) was used to stop the reaction. The absorbance was read at 450 nm wavelength and the concentration of IgA and IgG were reported as µg/mg of protein.

The OxiSelect Protein Carbonyl ELISA Kit (#STA-310, Cell Biolabs, Inc.; San Diego, CA, USA) was used to determine the concentration of protein carbonyl in the jejunal mucosa as previously described by Duarte and Kim [31]. The mucosal supernatant was diluted with PBS to get 10 µg/mL of protein to provide the concentration of protein carbonyl within the standard range (0.375 to 7.5 nmol/mg protein). The diluted samples and standards were pipetted in each well and incubate at 4 °C overnight. After washing, 100 µL dinitrophenylhydrazine (DNPH) working solution was added and incubate at room temperature for 45 min. Next, the plate was washed and 200 µL blocking solution was added following incubation with 1.5 h at room temperature on an orbital shaker. The anti-DNP antibody and was used after washing and the plate was incubated at room temperature for 1 h. The HRP conjugated secondary antibody was consecutively used after washing and the plate was incubated at room temperature for 1 h. Then 100 µL substrate solution was added in each well after washing and incubate at room temperature for 5 min. The reaction was stopped by adding 100 µL stop solution. The absorbance was measured at 450 nm wavelength and the concentration of protein carbonyl was reported as nmol/mg of protein. The OxiSelect TBARS MDA Quantitation Assay Kit (#STA-330, Cell Biolabs) was used to determine the concentration of MDA in the jejunal mucosa as previously described by Cheng et al. [25]. The samples and standards (100 µL) were added in separate 2-mL microcentrifuge tubes and SDS lysis solution (100 µL) and thiobarbituric acid (TBA) reagent (250 µL) were mix with samples and standards. Next, the tubes were placed in water bath at 95 °C for 1 h. The cooled tubes were centrifuged at 3,000 × *g* for 15 min to get the supernatant. The absorbance was read at 532 nm wavelength and the concentration of MDA was reported as nmol/mg of protein.

### Intestinal morphology and crypt cell proliferation

Mid-jejunal tissues of each pig were used to evaluate intestinal morphology and crypt cell proliferation. The tissues were kept in 10% buffered formaldehyde for 48 h for fixation. Two sections of fixed tissue (approximately 2 mm) were cut, placed in a cassette, and transferred to a 70% ethanol solution. The processed samples were sent to the North Carolina State University Histology Laboratory (College of Veterinary Medicine, Raleigh, NC, USA) for dehydration, embedment, and staining using a Ki-67 assay. The Biocare Intellipath Stainer (Biocare Medical, Pacheco, CA, USA) was used to process the automated Ki-67 staining. To get the proper working concentration, the primary monoclonal antibody of Ki-67 (#ACR325, Biocare Medical) was diluted (1:100), and then incubated for 30 min with processed slides at room temperature. For detection, Vector ImmPress Rabbit polymer was employed. Staining was carried out using chromogen diaminobenzidine (DAB). The microscope Olympus CX31 (Lumenera Corporation, Ottawa, Canada) and software (Infinity 2–2 digital CCD) were used to measure intestinal morphology (villus height, villus width, and crypt depth) at a magnification of 40× following the procedure described by Jang et al. [32]. Ten complete villi and crypts were chosen to represent the intestinal morphology of each pig. The villus length was measured from its top to its intersection with the crypt; the villus width was measured in the center of the villus; and the crypt depth was measured from its intersection with the villus to its bottom. The villus height was divided by the crypt depth to determine the villus height to crypt depth (VH:CD) ratio. The percentage of Ki-67 positive cells (the marker for proliferating cells in the crypt) was calculated using pictures of 10 complete crypts captured by the Olympus CX31 microscope at a magnification of 100× following the procedure described by Xu et al. [33]. The pictures were cropped and then uploaded to the software (Image JS) for analysis. All the procedures were conducted by the same person.

### Apparent ileal digestibility

Ileal digesta were placed in the freeze dryer (24D 48, Virtis, Gardiner, NY, USA) for 48 h for drying. The concentration of titanium dioxide in feed and digesta was measured following the method previously reported by Myers et al. [34]. The dry matter (DM) in feed and digesta was measured following Passos et al. [35]. The gross energy (GE) content in feed and digesta was measured using the bomb calorimeter (Parr 6200, Parr instrument company, Moline, IL, USA). The LECO CN-2000 Nitrogen Analyzer (LECO Corporation, St. Joseph, MI, USA) was used to measure the crude protein (CP) in feed and digesta. The apparent ileal digestibility (AID) of nutrients was calculated with the formula previously reported by Chen et al. [26]:

$$\mathrm{AID}={\{1\;-\;\lbrack({\mathrm{TiO}}_{2\mathrm{diet}}/{\mathrm{TiO}}_{2\mathrm{digesta}})\;\times\;({\mathrm{Nutrient}}_{\mathrm{digesta}}/{\mathrm{Nutrient}}_{\mathrm{diet}})\rbrack\}}\;\times100$$

In which TiO_2diet_ and TiO_2digesta_ represented the measured titanium dioxide concentration of diet and digesta, respectively; Nutrient_digesta_ and Nutrient_feed_ represented the measured nutrient concentration of the digesta and diet, respectively.

### Relative abundance and diversity of jejunal mucosa-associated microbiota

The QIAamp Fast DNA Stool Mini Kit (#51604, Qiagen, Germantown, MD, USA) was used to extract DNA from jejunal mucosas as previously described by Duarte and Kim [36]. The extracted DNA was sent to Diversigen Inc. (New Brighton, MN, USA) for shotgun metagenomic sequencing following their internal protocol. For BoosterShot (Diversigen: Shallow Sequencing, 5 million reads/sample), libraries were sequenced on an Illumina NovaSeq using paired-end 2 × 150 reads. The adaptor sequences were cut using Cutadapt, and the DNA sequences were screened based on quality (*Q*-Score < 30) and length (< 50). Using Bowtie2, host sequences were eliminated. The taxa tables were generated using the default parameters of the Kraken2 aligner (v2.0.8-beta). The database used was the “Standard” RefSeq database. The alignments were made at 97% identity against the reference genomes. In order to process further statistical analysis, the count data were converted to relative abundance as previously reported by Deng et al. [12] and Moita et al. [37]. The top 20 OTU data within each level were listed.

### Statistical analysis

Data were analyzed by the UNIVARIATE procedures (SAS Inst. Inc., Cary, NC, USA) to test for homogeneity, normality, and outliers. The MIXED procedure (SAS 9.4, SAS Inc.) was used to test the pre-planned contrasts. Dietary treatments were the main effect which was considered a fixed effect. Initial BW and sex were considered blocks and were added in the model as random effects. The experimental unit was the pig that was fed and housed individually. The pre-planned contrasts (CON vs. SPC, CON vs. ESB, CON vs. FSBL, and CON vs. FSBB) were conducted to determine the effects of each soy protein supplement replacing animal protein supplements. A *P* value less than 0.05 was considered statistically significant, and a *P* value between 0.05 and 0.10 was considered a tendency.

## Results

### Allergenic proteins in soy products

The concentrations of glycinin and β-conglycinin were reduced in soy protein concentrate, enzyme-treated soybean meal, and fermented soybean meal with *Bacillus* than soybean meal. However, the concentrate of glycinin and β-conglycinin in fermented soybean meal with *Lactobacillus* was greater than soybean meal. Compared to fermented soybean meal with *Bacillus*, soy protein concentrate and enzyme-treated soybean meal have lower concentration of glycinin and β-conglycinin (Table 1).

### Growth performance and fecal score

In this study, the SPC tended to decrease (*P* = 0.094) ADFI on overall. The ESB did not affect BW, ADG, ADFI, and G:F. The FSBL decreased (*P* < 0.05) BW on d 7 and 39 and tended to decrease (*P* = 0.050) BW on d 20. The FSBL decreased (*P* < 0.05) ADG in P1, and overall and tended to decrease (*P* = 0.078) ADG in P3. The FSBL decreased (*P* < 0.05) ADFI in P1, and P2 and tended to decrease (*P* = 0.095) ADFI on overall. The FSBB did not affect BW, ADG, ADFI, and G:F (Table 3).

**Table 3:** Growth performance of nursery pigs fed diets with SPC, enzyme-treated soy protein, and fermented soybean meal replacing animal protein supplements^a^

Item	Treatment					SEM	*P* value			
	CON	SPC^b^	ESB^b^	FSBL^b^	FSBB^b^		CON vs. SPC	CON vs. ESB	CON vs. FSBL	CON vs. FSBB
BW, kg										
d 0	6.6	6.6	6.6	6.6	6.6	0.3	0.862	0.914	0.948	0.931
d 7	7.3	7.1	7.1	6.8	7.2	0.2	0.394	0.370	0.035	0.592
d 20	12.0	11.3	11.5	11.0	11.6	0.4	0.177	0.389	0.050	0.414
d 39	26.1	24.6	25.0	24.1	25.3	0.8	0.122	0.230	0.038	0.381
ADG, g										
Phase 1 (d 0 to 7)	95	66	65	26	77	25	0.349	0.337	0.029	0.561
Phase 2 (d 7 to 20)	363	324	333	322	340	21	0.194	0.325	0.168	0.439
Phase 3 (d 20 to 39)	744	702	698	691	722	27	0.159	0.125	0.078	0.456
Overall (d 0 to 39)	500	462	458	449	479	18	0.116	0.208	0.036	0.375
ADFI, g										
Phase 1 (d 0 to 7)	141	116	118	92	131	16	0.212	0.260	0.017	0.605
Phase 2 (d 7 to 20)	494	438	456	410	463	29	0.170	0.359	0.043	0.452
Phase 3 (d 20 to 39)	1036	938	958	966	987	58	0.131	0.237	0.279	0.451
Overall (d 0 to 39)	695	624	641	624	659	36	0.094	0.215	0.095	0.395
G:F										
Phase 1 (d 0 to 7)	0.60	0.69	0.58	0.57	0.63	0.10	0.475	0.824	0.880	0.829
Phase 2 (d 7 to 20)	0.74	0.74	0.73	0.79	0.74	0.03	0.985	0.902	0.148	0.875
Phase 3 (d 20 to 39)	0.72	0.76	0.73	0.72	0.75	0.02	0.287	0.888	0.979	0.457
Overall (d 0 to 39)	0.72	0.75	0.73	0.73	0.74	0.02	0.447	0.967	0.973	0.618

^a^*n* = 12

^b^SPC, ESB, FSBL, and FSBB: basal diet with soy protein concentrate (X-Soy 200, CJ Selecta, Araguari, MG, Brazil), enzyme-treated soybean meal (HP 300, Hamlet Protein Inc., Findlay, OH, USA), fermented soybean meal with *Lactobacillus* (FerMex 200, Purina Animal Nutrition, Shoreview, SD, USA), and fermented soybean meal with *Bacillus* (Soytide, CJ Bio, Jung-gu, Seoul, Korea), replacing 1/3, 2/3, 3/3 of animal protein supplements in P1, P2, P3, respectively

There were no differences among the treatments on the fecal score during the entire experimental period (Table 4).

**Table 4:** Fecal score of nursery pigs fed diets with soy protein concentrate, enzyme processed soy protein, and fermented soybean meal replacing animal protein supplements^a^

Item	Treatment					SEM	*P* value			
	CON	SPC^b^	ESB^b^	FSBL^b^	FSBB^b^		CON vs. SPC	CON vs. ESB	CON vs. FSBL	CON vs. FSBB
Fecal score										
Phase 1 (d 0 to 7)	3.6	3.4	3.5	3.4	3.6	0.2	0.320	0.589	0.292	0.756
Phase 2 (d 7 to 20)	3.4	3.4	3.5	3.3	3.4	0.1	0.889	0.525	0.452	0.783
Phase 3 (d 20 to 39)	3.0	3.0	3.0	3.0	3.0	< 0.1	0.225	0.738	0.693	0.186

^a^*n* = 12

^b^SPC, ESB, FSBL, and FSBB: basal diet with soy protein concentrate (X-Soy 200, CJ Selecta, Araguari, MG, Brazil), enzyme-treated soybean meal (HP 300, Hamlet Protein Inc., Findlay, OH, USA), fermented soybean meal with *Lactobacillus* (FerMex 200, Purina Animal Nutrition, Shoreview, SD, USA), and fermented soybean meal with *Bacillus* (Soytide, CJ Bio, Jung-gu, Seoul, Korea), replacing 1/3, 2/3, 3/3 of animal protein supplements in P1, P2, P3, respectively

### Immune status and oxidative stress

The SPC did not affect the immune and oxidative stress parameters. The FSBL increased (*P* < 0.05) TNF-α content and tended to increase IgG (*P* = 0.089) and MDA (*P* = 0.065) content in the jejunal mucosa (Table 5). The FSBB tended to increase (*P* = 0.073) TNF-α content in the jejunal mucosa. The ESB tended to decrease (*P* = 0.098) the protein carbonyl content in the jejunal mucosa of weaned pigs compared to the CON treatment.

**Table 5:** Oxidative stress and immune status of nursery pigs fed diets with soy protein concentrate, enzyme-treated soy protein, and fermented soybean meal replacing animal protein supplements^a^

Item	Treatment						SEM	*P* value			
	CON		SPC^b^	ESB^b^	FSBL^b^	FSBB^b^		CON vs. SPC	CON vs. ESB	CON vs. FSBL	CON vs. FSBB
Jejunal mucosa											
IL-8^c^, pg/mg of protein	525	479		531	518	467	44	0.462	0.932	0.907	0.355
TNF-α^d^, pg/mg of protein	0.39	0.60		0.63	0.95	0.72	0.13	0.259	0.203	0.003	0.073
IgA^e^, µg/mg of protein	2.95	2.97		4.21	3.60	3.41	0.82	0.980	0.142	0.436	0.585
IgG^f^, µg/mg of protein	0.64	0.78		0.75	0.84	0.75	0.09	0.255	0.351	0.089	0.360
Protein carbonyl, nmol/mg of protein	1.93	1.59		1.38	1.56	1.60	0.34	0.290	0.098	0.247	0.300
MDA^g^, nmol/mg of protein	0.30	0.37		0.37	0.40	0.34	0.04	0.174	0.210	0.065	0.480

^a^*n* = 12

^b^SPC, ESB, FSBL and FSBB: basal diet with soy protein concentrate (X-Soy 200, CJ Selecta, Araguari, MG, Brazil), enzyme-treated soybean meal (HP 300, Hamlet Protein Inc., Findlay, OH, USA), fermented soybean meal with *Lactobacillus* (FerMex 200, Purina Animal Nutrition, Shoreview, SD, USA), and fermented soybean meal with *Bacillus* (Soytide, CJ Bio, Jung-gu, Seoul, Korea), replacing 1/3, 2/3, 3/3 of animal protein supplements in P1, P2, P3, respectively

^c^*IL-8* Interleukin 8

^d^*TNF-α* Tumor necrosis factor alpha

^e^*IgA* Immunoglobulin A

^f^*IgG* Immunoglobulin G

^g^*MDA* Malondialdehyde

### Intestinal morphology and crypt cell proliferation

There were no differences in villus height, villus width, crypt depth, and VH:CD ratio among the treatments. The SPC tended to increase (*P* = 0.091) the ratio of Ki-67 positive cells to total cells compared to CON (Table 6).

**Table 6:** Intestinal morphology of nursery pigs fed diets with soy protein concentrate, enzyme-treated soy protein, and fermented soybean meal replacing animal protein supplements^a^

Item	Treatment					SEM	*P* value			
	CON	SPC^b^	ESB^b^	FSBL^b^	FSBB^b^		CON vs. SPC	CON vs. ESB	CON vs. FSBL	CON vs. FSBB
Villus height, µm	510	472	484	527	485	30	0.366	0.537	0.693	0.545
Villus width, µm	118	114	116	115	115	3	0.398	0.650	0.495	0.471
Crypt depth, µm	285	272	277	287	275	9	0.326	0.562	0.858	0.455
VH:CD^c^	1.84	1.73	1.76	1.84	1.78	0.13	0.522	0.645	0.984	0.725
Ki-67 positive, %	26.7	29.0	28.3	27.8	26.1	1.7	0.091	0.239	0.383	0.676

^a^*n* = 12

^b^SPC, ESB, FSBL, and FSBB: basal diet with soy protein concentrate (X-Soy 200, CJ Selecta, Araguari, MG, Brazil), enzyme-treated soybean meal (HP 300, Hamlet Protein Inc., Findlay, OH, USA), fermented soybean meal with *Lactobacillus* (FerMex 200, Purina Animal Nutrition, Shoreview, SD, USA), and fermented soybean meal with *Bacillus* (Soytide, CJ Bio, Jung-gu, Seoul, Korea), replacing 1/3, 2/3, 3/3 of animal protein supplements in P1, P2, P3, respectively

^c^*VH:CD *Villus height to crypt depth ratio

### Apparent Ileal digestibility

The SPC, ESB, and FSBB did not affect the AID of DM, GE, and CP compared to CON. The FSBL increased (*P* < 0.05) AID of CP (Table 7).

**Table 7:** Apparent ileal digestibility of nutrients of nursery pigs fed diets with soy protein concentrate, enzyme-treated soy protein, and fermented soybean meal replacing animal protein supplements^a^

Item	Treatment					SEM	*P* value			
	CON	SPC^b^	ESB^b^	FSBL^b^	FSBB^b^		CON vs. SPC	CON vs. ESB	CON vs. FSBL	CON vs. FSBB
DM^c^, %	50.7	46.4	54.5	56.4	50.0	3.1	0.367	0.381	0.188	0.884
GE^d^, %	51.0	47.0	55.6	57.6	51.2	3.3	0.408	0.318	0.144	0.960
CP^e^, %	57.6	55.2	61.7	68.5	57.6	3.2	0.600	0.369	0.016	0.995

^a^*n* = 12

^b^SPC, ESB, FSBL, and FSBB: basal diet with soy protein concentrate (X-Soy 200, CJ Selecta, Araguari, MG, Brazil), enzyme-treated soybean meal (HP 300, Hamlet Protein Inc., Findlay, OH, USA), fermented soybean meal with *Lactobacillus* (FerMex 200, Purina Animal Nutrition, Shoreview, SD, USA), and fermented soybean meal with *Bacillus* (Soytide, CJ Bio, Jung-gu, Seoul, Korea), replacing 1/3, 2/3, 3/3 of animal protein supplements in P1, P2, P3, respectively

^c^*DM* Dry matter

^d^*GE* Gross energy

^e^*CP* Crude protein

### Relative abundance and diversity of jejunal mucosa-associated microbiota

At the phylum level, the replacement of soy products did not affect the relative abundance of jejunal mucosa-associated microbiota (Table 8).

**Table 8:** Relative abundance of jejunal mucosa-associated microbiota at the phylum level in nursery pigs fed diets with soy protein concentrate, enzyme-treated soy protein, and fermented soybean meal replacing animal protein supplements^a^

Item	Treatment					SEM	*P* value			
	CON	SPC^b^	ESB^b^	FSBL^b^	FSBB^b^		CON vs. SPC	CON vs. ESB	CON vs. FSBL	CON vs. FSBB
Proteobacteria	44.52	42.89	43.99	42.05	41.40	2.55	0.435	0.802	0.240	0.140
Firmicutes	30.37	32.39	29.51	32.04	34.12	3.89	0.378	0.706	0.467	0.106
Actinobacteria	14.41	14.74	15.71	15.28	13.93	2.01	0.863	0.502	0.652	0.803
Bacteroidetes	3.89	3.10	3.25	3.57	3.53	0.39	0.398	0.650	0.495	0.471
Others	6.81	6.88	7.53	7.07	7.02	0.49	0.919	0.275	0.691	0.749

^a^*n* = 12

^b^SPC, ESB, FSBL, and FSBB: basal diet with soy protein concentrate (X-Soy 200, CJ Selecta, Araguari, MG, Brazil), enzyme-treated soybean meal (HP 300, Hamlet Protein Inc., Findlay, OH, USA), fermented soybean meal with *Lactobacillus* (FerMex 200, Purina Animal Nutrition, Shoreview, SD, USA), and fermented soybean meal with *Bacillus* (Soytide, CJ Bio, Jung-gu, Seoul, Korea), replacing 1/3, 2/3, 3/3 of animal protein supplements in P1, P2, P3, respectively

At the genus level, SPC tended to increase (*P* = 0.065) the relative abundance of *Staphylococcus*. The FSBL tended to increase (*P* = 0.078) the relative abundance of *Klebsiella.* The FSBB increased (*P* < 0.05) the relative abundance of *Clostridium* and decreased (*P* < 0.05) the relative abundance of *Achromobacter*. The FSBB tended to increase (*P* = 0.074) the relative abundance of *Pseudomonas* and tended to decrease (*P* = 0.078) the relative abundance of *Nocardia* (Table 9).

**Table 9:** Relative abundance of jejunal mucosa-associated microbiota at the genus level in nursery pigs fed diets with soy protein concentrate, enzyme-treated soy protein, and fermented soybean meal replacing animal protein supplements^a^

Item	Treatment					SEM	*P* value			
	CON	SPC^b^	ESB^b^	FSBL^b^	FSBB^b^		CON vs. SPC	CON vs. ESB	CON vs. FSBL	CON vs. FSBB
*Clostridium*	13.44	12.74	13.06	14.13	16.95	2.92	0.684	0.827	0.688	0.048
*Staphylococcus*	10.47	12.32	10.87	10.67	9.74	0.69	0.065	0.688	0.841	0.453
*Sphingomonas*	8.04	6.12	7.49	6.17	6.10	1.26	0.137	0.667	0.147	0.133
*Streptomyces*	6.53	8.08	8.40	7.56	7.75	1.41	0.371	0.282	0.550	0.479
*Agrobacterium*	4.84	5.19	3.69	2.93	5.01	1.32	0.843	0.520	0.287	0.923
*Schlegelella*	3.61	3.16	3.91	3.46	2.82	0.56	0.365	0.545	0.765	0.114
*Pseudomonas*	2.73	2.96	3.23	2.86	3.28	0.30	0.435	0.104	0.657	0.074
*Pseudoalteromonas*	2.72	2.84	2.69	3.07	3.01	0.22	0.695	0.930	0.274	0.364
*Achromobacter*	2.02	1.69	2.01	1.83	1.31	0.23	0.306	0.955	0.542	0.032
*Klebsiella*	1.95	2.02	1.76	2.77	1.67	0.37	0.862	0.679	0.078	0.550
*Salmonella*	1.88	1.81	1.96	1.88	1.08	0.21	0.816	0.774	0.995	0.802
*Deinococcus*	1.83	1.88	2.01	1.92	1.73	0.16	0.802	0.392	0.668	0.623
*Xanthomonas*	1.74	2.26	2.01	1.83	1.56	0.28	0.168	0.468	0.820	0.622
*Bacillus*	1.59	1.63	1.35	1.69	1.72	0.34	0.914	0.447	0.764	0.683
*Micromonospora*	1.26	0.84	1.33	1.04	0.92	0.31	0.125	0.787	0.416	0.205
*Nocardia*	1.23	1.34	0.90	1.15	0.67	0.22	0.725	0.298	0.788	0.078
*Leptospira*	1.22	0.81	1.27	0.96	0.58	0.43	0.346	0.911	0.534	0.144
*Mycolicibacterium*	1.09	0.74	1.05	0.96	1.08	0.16	0.101	0.843	0.522	0.975
*Mycobacterium*	0.98	0.88	0.93	1.10	0.84	0.15	0.599	0.790	0.519	0.457
*Lelliottia*	0.93	0.75	0.84	0.91	0.78	0.13	0.296	0.590	0.897	0.384
Others	29.91	29.93	29.25	31.14	30.69	1.11	0.925	0.727	0.579	0.599

^a^*n* = 12

^b^SPC, ESB, FSBL, and FSBB: basal diet with soy protein concentrate (X-Soy 200, CJ Selecta, Araguari, MG, Brazil), enzyme-treated soybean meal (HP 300, Hamlet Protein Inc., Findlay, OH, USA), fermented soybean meal with *Lactobacillus* (FerMex 200, Purina Animal Nutrition, Shoreview, SD, USA), and fermented soybean meal with *Bacillus* (Soytide, CJ Bio, Jung-gu, Seoul, Korea), replacing 1/3, 2/3, 3/3 of animal protein supplements in P1, P2, P3, respectively

At the species level, SPC tended to increase (*P* = 0.069) the relative abundance of *Staphylococcus aureus*. The FSBL tended to decrease (*P* = 0.096) the relative abundance of *Sphingomonas* sp. Cra20. The FSBB increased (*P* < 0.05) the relative abundance of *Clostridium botulinum*, and tended to decrease (*P* = 0.063) the relative abundance of *Achromobacter spanius* (Table 10).

**Table 10:** Relative abundance of jejunal mucosa-associated microbiota at the species level in nursery pigs fed diets with soy protein concentrate, enzyme-treated soy protein, and fermented soybean meal replacing animal protein supplements^a^

Item	Treatment					SEM	*P* value			
	CON	SPC^b^	ESB^b^	FSBL^b^	FSBB^b^		CON vs. SPC	CON vs. ESB	CON vs. FSBL	CON vs. FSBB
*Clostridium botulinum*	11.08	10.42	10.83	11.64	14.21	2.48	0.664	0.868	0.707	0.042
*Staphylococcus aureus*	10.34	12.18	10.75	10.53	9.52	0.70	0.069	0.675	0.843	0.410
*Sphingomonas* sp. AAP5	6.39	4.84	6.11	5.16	4.86	1.08	0.183	0.808	0.290	0.190
*Agrobacterium fabrum*	4.84	5.19	3.69	2.93	5.01	1.32	0.843	0.520	0.287	0.923
*Schlegelella thermodepolymerans*	3.61	3.16	3.91	3.46	2.82	0.56	0.365	0.545	0.765	0.114
*Streptomyces platensis*	2.04	2.14	2.49	1.90	1.73	0.49	0.792	0.253	0.731	0.440
*Salmonella enterica*	1.88	1.81	1.96	1.88	1.80	0.21	0.816	0.774	0.995	0.802
*Deinococcus deserti*	1.83	1.88	2.01	1.92	1.73	0.16	0.802	0.392	0.668	0.623
*Achromobacter spanius*	1.80	1.43	1.79	1.55	1.20	0.24	0.247	0.974	0.427	0.063
*Streptomyces mobaraensis*	1.74	1.80	2.57	1.88	1.80	0.54	0.909	0.137	0.788	0.910
*Sphingomonas* sp. Cra20	1.65	1.28	1.38	1.01	1.24	0.27	0.333	0.478	0.096	0.280
*Pseudoalteromonas atlantica*	1.55	1.37	1.35	1.91	1.78	0.17	0.474	0.427	0.151	0.354
*Micromonospora auratinigra*	1.26	0.84	1.33	1.04	0.92	0.31	0.125	0.787	0.416	0.205
*Leptospira kmetyi*	1.22	0.81	1.27	0.96	0.58	0.43	0.346	0.911	0.534	0.144
*Pseudoalteromonas prydzensis*	1.17	1.47	1.34	1.16	1.23	0.19	0.261	0.527	0.975	0.819
*Pseudomonas stutzeri*	1.04	0.86	0.92	0.86	1.07	0.11	0.238	0.432	0.256	0.860
*Klebsiella pneumoniae*	1.01	1.03	0.72	1.70	0.58	0.33	0.967	0.518	0.138	0.342
*Lelliottia amnigena*	0.93	0.75	0.84	0.91	0.78	0.13	0.296	0.590	0.897	0.384
*Methylocaldum marinum*	0.90	1.06	0.79	1.25	0.84	0.30	0.707	0.800	0.423	0.889
*Klebsiella variicola*	0.78	0.81	0.87	0.90	0.87	0.14	0.818	0.516	0.379	0.482
Others	42.96	44.86	43.08	45.46	45.43	1.26	0.554	0.862	0.171	0.600

^a^*n* = 12

^b^SPC, ESB, FSBL, and FSBB: basal diet with soy protein concentrate (X-Soy 200, CJ Selecta, Araguari, MG, Brazil), enzyme-treated soybean meal (HP 300, Hamlet Protein Inc., Findlay, OH, USA), fermented soybean meal with *Lactobacillus* (FerMex 200, Purina Animal Nutrition, Shoreview, SD, USA), and fermented soybean meal with *Bacillus* (Soytide, CJ Bio, Jung-gu, Seoul, Korea), replacing 1/3, 2/3, 3/3 of animal protein supplements in P1, P2, P3, respectively

At the alpha diversity, FSBB decreased (*P* < 0.05) Simpson index at the genus and species level (Table 11). There were no differences in SPC, ESB and FSBL compared to CON treatment on the alpha diversity of the mucosa-associated microbiota in jejunum.

**Table 11:** Alpha diversity of jejunal mucosa-associated microbiota estimated with Chao1 richness, Shannon diversity, and Simpson diversity in nursery pigs fed diets with soy protein concentrate, enzyme-treated soy protein, and fermented soybean meal replacing animal protein supplements^a^

Item	Treatment					SEM	*P* value			
	CON	SPC^b^	ESB^b^	FSBL^b^	FSBB^b^		CON vs. SPC	CON vs. ESB	CON vs. FSBL	CON vs. FSBB
Genus										
Chao1	111.63	109.66	107.85	110.26	109.25	2.11	0.471	0.171	0.614	0.385
Shannon	3.64	3.61	3.60	3.69	3.58	0.03	0.480	0.320	0.288	0.113
Simpson	0.95	0.94	0.94	0.95	0.93	<0.01	0.566	0.571	0.727	0.005
Species										
Chao1	165.96	164.01	164.33	164.17	167.29	2.19	0.428	0.507	0.466	0.589
Shannon	4.10	4.09	4.08	4.17	4.06	0.03	0.971	0.756	0.155	0.399
Simpson	0.96	0.96	0.96	0.96	0.95	<0.01	0.697	0.938	0.533	0.026

^a^*n* = 12

^b^SPC, ESB, FSBL, and FSBB: basal diet with soy protein concentrate (X-Soy 200, CJ Selecta, Araguari, MG, Brazil), enzyme-treated soybean meal (HP 300, Hamlet Protein Inc., Findlay, OH, USA), fermented soybean meal with *Lactobacillus* (FerMex 200, Purina Animal Nutrition, Shoreview, SD, USA), and fermented soybean meal with *Bacillus* (Soytide, CJ Bio, Jung-gu, Seoul, Korea), replacing 1/3, 2/3, 3/3 of animal protein supplements in P1, P2, P3, respectively

## Discussion

In this study, 1/3, 2/3, and 3/3 animal protein supplements in diets for nursery pigs were replaced by different soy protein supplements in phase 1, 2, and 3, separately. Soy protein concentrate, enzyme-treated soybean meal, and fermented soybean meal with *Bacillus* showed the potential to partly replace animal protein supplements without affecting growth performance, whereas fermented soybean meal with *Lactobacillus* replacing animal protein supplements in diets for nursery pigs impaired growth performance and intestinal health. The negative effects may be associated with the high level of allergenic proteins in fermented soybean meal with *Lactobacillus*.

Antinutritional factors in soybean, such as trypsin inhibitors, lectins, and soy allergenic proteins, limit its use in nursery diets due to the adverse effects on growth performance of nursery pigs [38, 39]. In particular, glycinin and β-conglycinin are two soy antigens predominantly found in soybean meal resistant to heat processing, which can result in hypersensitive reaction and then impair growth performance of nursery pigs [40, 41]. Several antigenic epitopes were identified in glycinin and β-conglycinin [42–44]. In this study, ethanol extraction and enzymatic hydrolysis could efficiently reduce the concentration of allergenic proteins in soybean meal, which was in accordance with the previous studies [11, 12]. However, the fermentation of soybean meal remained a relatively higher concentration of allergenic proteins compared to ethanol extraction and enzymatic hydrolysis. In general, the quality of fermentation related to reducing the allergenicity in soybean meal can be mainly attributed to the fermentation process, which includes temperature, duration, pH, and microorganisms used in the process [8]. *Bacillus subtilis* and *Lactobacillus plantarum*, two commonly used bacterial strains for fermentation of soybean meal, were used in this study [45, 46]. Due to the different protease profiles and secretion abilities from these microorganisms, the fermented soybean meal may have varied contents of allergenic proteins, which could explain the variation between two fermented soybean meals used in this study [8]. Consequently, the relatively high allergenic proteins in fermented soybean meal can potentially impair growth performance of nursery pigs [47].

In this study, SPC partly replacing animal protein supplements in nursery diets did not affect the growth performance of pigs, which was in accordance with a previous study [12]. This could be associated with low allergenic proteins (less than 0.1 mg/g glycinin and 0.1 mg/g β-conglycinin) and balanced amino acid profile in soy protein concentrate. Even though the fermented soybean meal was indicated to a high digestible protein source, the growth performance of pigs in FSBL was impaired during the entire experiment [48]. Firstly, it can be explained by increased soy allergenic proteins in the diets. According to Friesen et al. [49], the inclusion of soybean meal containing high allergenic proteins in the diets of early-weaned pigs would decrease the growth performance. Secondly, it can be explained by the reduction of animal protein supplements in diets. Blood plasma has been indicated positive effects to stimulate the feed intake of nursery pigs [50]. Also, previous studies have shown that fish meal and poultry meal have the potential to stimulate feed intake of pigs [5, 51]. With the replacement of appetitive animal protein supplements by fermented soybean meal with *Lactobacillus*, the feed intake of nursery pigs can be negatively affected. In current study, no difference in fecal score were observed. Although, the allergenic proteins in soybean meal may result in diarrhea through interfering intestinal immune responses [52], the calculated concentration of allergenic proteins in soy protein concentrate and enzyme-treated soybean meal were similar to the control diet. Interestingly, fermented soybean meal with *lactobacillus* containing high allergenic proteins did not affect fecal score in this study. This result might be explained by the presence of *Lactobacillus*. Previous study has been indicated that *Lactobacillus* could improve function of the intestinal barrier then effectively prevent post-weaning diarrhea [53]. Even though the relatively higher allergenic proteins in FSBL, *Lactobacillus* may play some roles in preventing diarrhea.

Intestinal immunity is one of the most essential factors in pigs that is highly responsible for growth performance [54]. In this study, pigs in FSBL increased the content of MDA, IgG, and TNF-α in jejunal mucosa compared to CON treatment. The MDA, one of the major products of lipid peroxidation, can be a direct indicator of lipid oxidative damage in the body [55]. Ma et al. [56] indicated that the weaned pigs fed diets containing low soy allergenic proteins decreased MDA concentration in serum, which could be contributed to the increased glutathione peroxidase (GSH-Px) activity. The IgG and TNF-α are also important biomarkers for pathogen infections and health status of the host [57, 58]. Pigs exposed to soy antigens increased the level of IgG due to the hypersensitivity reactions in pigs, which was highly associated with diarrhea [59–61]. The increased TNF-α in FSBL can also be explained by the high level of soy antigens. Peng et al. [62] suggested that soy antigens could increase the level of TNF-α through their impact on the expression of nuclear factor-kappa B (NF-κB), p38, and Jun N-terminal kinase. The FSBB also increased the level of TNF-α, which might be associated with relatively high level of antigens. The increased oxidative stress and immune response in the GI tract of nursery pigs indicated that soy antigens in fermented soybean meal severely impaired the intestinal integrity, then possibly resulted in the impaired growth performance [63].

Intestinal morphology reflects the nutrient digestion and absorption capacity of pigs [64]. Antinutritional factors in soybean meal, such as trypsin inhibitor (TI), have been shown to negatively affect the intestinal morphology of newly weaned pigs due to the interference with trypsin and chymotrypsin [9, 65]. In this study, no differences in intestinal morphology were observed among treatments, which indicated the different processes of soybean meal could efficiently remove the antinutritional factor in soybean meal. Feng et al. [66] showed that the fermentation of soybean meal with *Bacillus subtilis* could completely remove trypsin inhibitor in soybean meal. Enzyme treatment and ethanol extraction were indicated to partly remove TI in soybean meal [11, 67]. These findings are in accordance with the results of the current study. Nutrient digestibility is highly related to intestinal morphology. Due to the unchanged intestinal morphology among treatments, the nutrient digestibility was not affected in SPC, ESB, and FSBB. However, FSBL increased the crude protein digestibility, which can be attributed to the supplemental effect of *Lactobacillus*. Lactic acid and proteolytic enzymes produced by *Lactobacillus* can improve the nutrition digestibility in the GI tract [68]. Previous studies showed that the inclusion of *Lactobacillus* or *Lactobacillus* metabolites in nursery diets could enhance the crude protein digestibility of pigs, which was in accordance with the current study [69, 70].

It has been demonstrated that changing the protein source in the diets can modulate the microbiota composition of pigs because it changes the physicochemical conditions and the substrate availability in the intestine [12, 71]. In this study, FSBB increased the relative abundance of *Clostridium* and decreased the relative abundance of *Achromobacter*. Fermented soybean meal containing *Bacillus* and its metabolites may change the environment in the intestine, which could have promoted the growth of Firmicute bacteria including *Clostridium*, and correspondingly have reduced the growth of Proteobacteria including *Achromobacter*. Fermented soybean meal containing *Lactobacillus* did not affect the abundance of genera belonging to the Firmicutes, however, it increased the relative abundance of *Klebsiella*, a Proteobacteria that is considered a harmful bacteria causing different infections in animals and humans [72]. The increased relative abundance of *Klebsiella* can be associated with the increased intestinal immune response and oxidative stress of pigs in this study. In addition, FSBB decreased the alpha diversity of jejunal mucosa-associated microbiota in this study. The previous study showed that diet complexity could positively correlate with the microbiota diversity of pigs [73]. When all animal protein supplements were replaced by fermented soybean meal with *Bacillus* in P3, the diet complexity decreased, and then decreased the microbiota diversity. This result corresponded with the study previously reported by Deng et al. [12].

## Conclusion

Soy protein concentrate, enzyme-treated soybean meal, and fermented soybean meal with *Bacillus* could reduce the use of animal protein supplements up to 33% until 7 kg body weight, up to 67% from 7 to 11 kg body weight, and entirely from 11 kg body weight without affecting the intestinal health and the growth performance of nursery pigs. However, fermented soybean meal with *Lactobacillus* partly replacing animal protein supplements reduced the growth performance of nursery pigs, which was contributed by increasing the immune reaction and oxidative stress in the intestine.

## Data Availability

All data generated or analyzed during this study are available from the corresponding author upon reasonable request.

## Abbreviations

AA: Amino acid
ADFI: Average daily feed intake
ADG: Average daily gain
AID: Apparent ileal digestibility
BW: Body weight
CP: Crude protein
DM: Dry matter
DNA: Deoxyribonucleic acid
ELISA: Enzyme-linked immunosorbent assay
GE: Gross energy
GF: Gain to feed ratio
MDA: Malondialdehyde
IgA: Immunoglobulin A
IgG: Immunoglobulin G
IL-8: Interleukin-8
OUTs: Operational taxonomic units
PBS: Phosphate-buffered saline
SID: Standard ileal digestibility
TI: Trypsin inhibitor
TiO_2_: Titanium dioxide
TNF-α: Tumor necrosis factor-alph
VH:CD: Villus height to crypt depth ratio
